# PASS2 version 6: a database of structure-based sequence alignments of protein domain superfamilies in accordance with SCOPe

**DOI:** 10.1093/database/baz028

**Published:** 2019-03-01

**Authors:** Pritha Ghosh, Teerna Bhattacharyya, Oommen K Mathew, Ramanathan Sowdhamini

**Affiliations:** National Centre for Biological Sciences, Tata Institute of Fundamental Research, Bellary Road, Bangalore, Karnataka, India

## Abstract

The number of protein structures is increasing due to the individual initiatives and rapid development of structure determination techniques. Structure-based sequence alignments of distantly related proteins enable the investigation of structural, evolutionary and functional relationships between proteins and their domains leading to their common evolutionary origin. Protein Alignments organized as Structural Superfamilies (PASS2) is a database that provides such alignments of members of protein domain superfamilies of known structure and with less than 40% sequence identity. PASS2 has been continuously updated in accordance to Structural Classification of Proteins (SCOP), and now Structural Classification of Proteins - extended (SCOPe). The current update directly corresponds to SCOPe 2.06, dealing with 2006 domain superfamilies of known structure and about 14 000 domains. Alignments have been augmented by features such as hidden Markov models, highly conserved residues, structural motifs and gene ontology terms, which are available for download. In this update, we introduce the concepts of ‘extreme structural outliers’ and ‘split superfamilies’ as well.

## Introduction

With the rapid growth of structure determination methods, the number of protein structures deposited in the Protein Data Bank (PDB) has been growing exponentially. This provides us with rich data to understand more about the protein structure–function relationships. However, it is important to classify the structures according to their evolutionary origin. For proteins of known structure, the Structural Classification of Proteins (SCOP) database provides comprehensive description of the structural and evolutionary relationships. Family and superfamily levels denote the close and distant evolutionary relationships between structural domains, respectively. Domains within the same fold hierarchy retain mere structural similarities that may not have a bearing on their biological functions. SCOPe extends SCOP through a combination of automation and manual curation (1).

Sequence alignments of proteins are of key importance in understanding structural, evolutionary and functional relationships between proteins. Proteins descending from the same common ancestor and falling within the same superfamily can, however, be sequentially divergent, thus rendering routine sequence alignment methods inappropriate. In such cases, structure-based sequence alignments of superfamily members serve as a guiding evolutionary model, which can be used for the identification of conserved residues or motifs, modelling of distant homologues, genome-wide sequence searches as well as genome-wide association studies leading to single nucleotide polymorphism (SNP) identification and drug discovery (2, 3).

Protein Alignments organized as Structural Superfamilies (PASS2) is a database that provides structure-based sequence alignments for protein domain superfamilies and has been updated continuously since 2002 (2). Protein structural domains within a superfamily that have less than 40% sequence identity among themselves are recognized from SCOPe and considered for structure-based sequence alignment and subsequent annotation in PASS2. The sequence identity filter aids to avoid redundancy and, hence, reduces computational time required for the rigorous alignment protocol (4). It also enables us to avoid alignments between closely related protein domains, which can be achieved by automatic multiple sequence alignment algorithms. The alignment produced for each superfamily is annotated and systematically included in the PASS2 database. The current update, PASS2.6, is in accordance with SCOPe 2.06, where we have assembled structure-based sequence alignments of 2006 superfamilies with close to 14 000 structures of protein domains. Features, such as solvent accessibility, Hidden Markov Model (HMM) profiles, absolute and highly conserved residues (HCRs), have also been provided alongside the alignments. We have modified the pipeline in order to achieve higher automation. We also discuss how outliers and extreme outliers were handled, where alignment of all structural domains within a superfamily proved challenging. Finally, a few case studies have been discussed for exemplification.

## Materials and methods

Structural domains, with no more than 40% sequence identity with each other, were obtained from ASTRAL 2.06 (corresponding to SCOPe 2.06) and these were organized into superfamilies. The superfamilies were then classified on the basis of the number of constituent domains into single-member superfamilies (SMS) and multi-member superfamilies (MMS). For this update, several scripts (Python and Perl) were included in the pipeline to identify and remove incomplete residues, remove ‘GLX’ (GLU/GLN ambiguous) and ‘UNK’ (Unknown) characters from PDB files before carrying out initial alignment.

Protein domains are denoted by the 7-character SCOPe ID [which is referred by the stable domain identifier (sid): ‘d’ followed by the 4-character PDB ID from which the file is derived, the PDB chain ID (none: ‘_’, multiple chains: ‘.’), and an integer to specify the domain uniquely]. For example, d2nn6e1 and d2nn6e2 refers to SCOPe domains with residues 5–191 and 192–285 of chain E from the PDB ID 2NN6, respectively. Individual domains in such multi-domain proteins are considered independent of each other. Hence, a protein can be classified to one or more SCOPe superfamilies, based on the SCOPe classification of its constituent domains. Thus, proteins with evolutionarily-related constituent domains are classified to the same SCOPe superfamily, whereas those in which the constituent domains are not evolutionarily related (and hence have divergent structure and function) are classified to two or more SCOPe superfamilies.

Initial alignment of the structural domains within each superfamily was obtained using Matt (Multiple Alignment with Translations and Twists) (5), which also provides a structural distance-based phylogenetic tree. The JOY program (6) is used for the annotation of structural features (secondary structural elements, solvent accessibility of residues, hydrogen bonding pattern, etc.) of the protein domains and using JOY, the initial alignment was also annotated with structural features. JOY was used to identify equivalences in the initial alignment—non-gapped aligned regions in each member of the superfamily. The structure-guided tree and equivalences were provided as inputs for COMPARER (7), which uses variable gap penalties and local structural features (such as backbone conformation, solvent accessibility and hydrogen bonding patterns) to create the final structure-based sequence alignment. In general, the variable gap penalties ensure that there are no unreasonable gaps in between secondary structures and conserved regions within the alignment. MNYFIT (6) was used for rigid-body superposition of the structures and it required equivalences as input, which were extracted from the final alignment using JOY. The MNYFIT program derives a protein framework defined as a weighted average of topologically equivalent positions, but without a recognized geometry (8). It uses the least squares fitting algorithm of McLachlan (9), and performs a rigid-body superposition of the C^α^ backbones of two or more protein structures using equivalent residues.

Although members of a superfamily are expected to be structurally similar or have a common fold, we came across cases where one or more domain(s) in the superfamily would be structurally deviant; these would either have more than 5.5 Å root mean square deviation (RMSD) with other members (structural outliers) or fail to align with any other member (extreme structural outliers) during COMPARER alignment (10, 11). Such domains were removed from the list and other files, using a Python script, and the remaining domains were realigned. We also encountered a few examples where JOY failed to extract equivalences from the COMPARER-derived alignment of members of a superfamily. In such cases, this alignment and the RMSD-based structural phylogeny can be used as a reference to split the superfamily and obtain superposed structures of the subgroups within the superfamily (split superfamily). The members of each split superfamily may or may not correspond to same structural families (a hierarchical level lower than the structural superfamilies) as defined by SCOPe.

As in the previous version of PASS2, HMMs of alignments of superfamily members have been created using hmmbuild module of HMMER suite (12, 13). Conserved secondary structural motifs have also been recorded using in-house SMotif program (14). Further, in order to recognize likely functional motifs, HCRs and ‘absolutely conserved residues (ACRs) were extracted from the alignments of all superfamilies, where 80–99% and 100% amino acid conservation is resident at such alignment positions, respectively.

The domains were annotated with JOY to produce accessory files such as PSA (solvent accessibility), HBD (hydrogen bond networks) and SST (secondary structures). The alignment was also annotated with JOY to provide information about solvent accessibility, hydrogen bonding patterns and secondary structural elements, and the annotated alignment is available for download by the user. Principal component analysis (PCA) plots have been constructed on the basis of sequence dissimilarity distribution of members of a superfamily and are available for download by the users. Moreover, alignment statistics (ALISTAT) and indel information (CUSP) have been provided for each superfamily (15). C^α^ RMSDs, at structurally equivalent positions of members of each superfamily, were used to construct structure-guided trees (structure-based phylogeny). The C^α^ RMSD values, as well as such tree files, are also available for user download. Gene ontology (GO) represents properties of gene product under three major terms, namely cellular component, molecular function and biological process (16). GO term(s), corresponding to each member within superfamilies, were retrieved dynamically from www.rcsb.org using the RestFul API clients written in Python.

MySQL 5.2 was employed as database engine for this version, along with Python2.7 and BioPython (17) for back-end data retrieval implementation and manipulation logic. The user interface was built using the components from HTML5, CSS, JavaScript, Ajax and JQuery. The visualization of the molecular structures and phylogenetic tree has been implemented using JSMol and raphael and jsPhyloSVG (18). The visualization of the alignment and corresponding HCRs and ACRs have been implemented using an in-house plug-in.

## Results

### Statistics of the updated database

**Figure 1:**
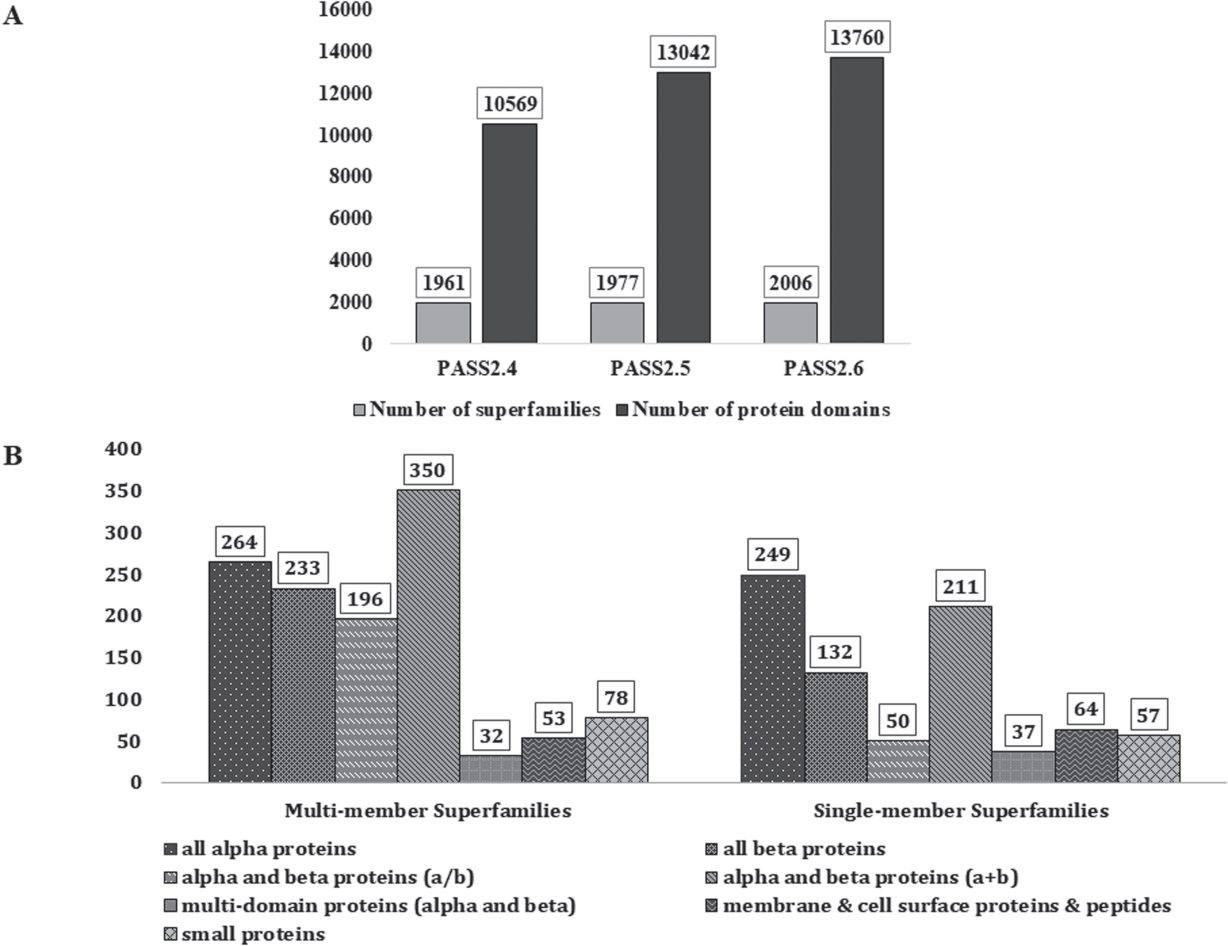
PASS2 statistics. (**A**) Statistics and comparison of current update with two earlier versions of PASS2, in terms of number of superfamilies (light grey) and number of protein domains included (dark grey). (**B**) Distribution of SCOPe structural classes (all α, all β, α/β and so on) in single and MMS considered for alignment in PASS2.6.

In PASS2.6, 2006 superfamilies were considered, out of which 1206 were found to be MMS and 800 had only one structural domain (SMS). A total of 13 760 protein domains, belonging to 2006 superfamilies, have been considered for this update. Figure 1A represents the increment in number of MMS and number of domains over the last few updates of PASS2 database. The superfamilies considered for this update belong to the seven classes (a–g) of protein domains in SCOPe 2.06 and their distribution is seen in Figure 1B. The MMS within this version of PASS2 mostly adopt alpha and beta proteins (a/b) or all-alpha proteins. However, a vast majority of SMS belong to the structural class of all alpha-proteins, followed by alpha and beta proteins (a/b).

### Modification of pipeline and case studies

**Figure 2:**
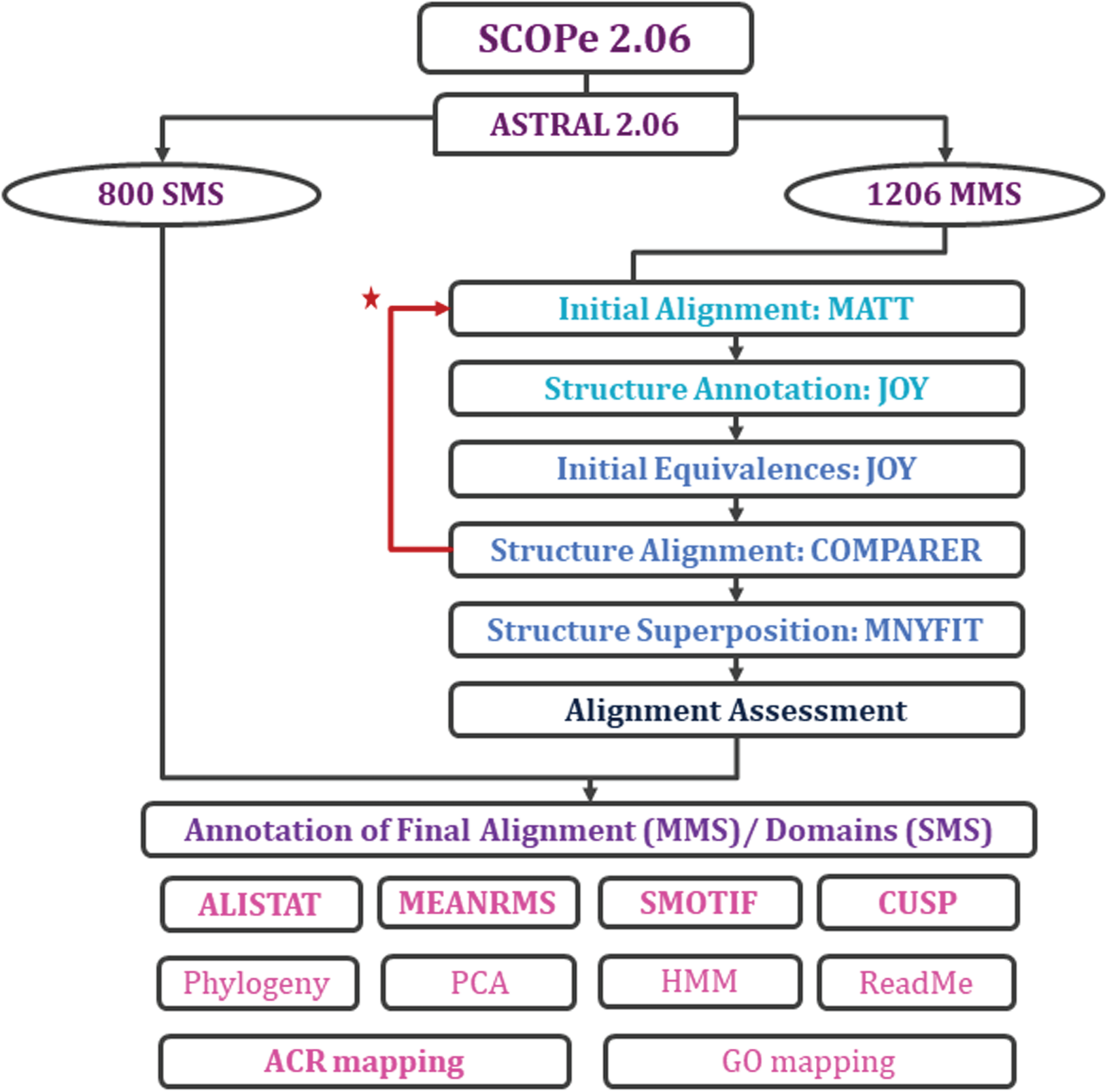
Workflow for rigorous structure-based sequence alignment of protein domains with less than 40% sequence identity belonging to SCOPe superfamilies. The initial alignment, final alignment and alignment assessment phases are coloured in cyan, blue and indigo. Features that were generated for the alignments (for MMS) and domains (for SMS) are listed, with in-house features in bold. Extreme structural outliers were removed from superfamilies if required and the remaining members were realigned (marked by red star).

With each update of PASS2 database, the aim has been better automation of the rigorous protocol (Figure 2 and Supplementary Figure 1) of alignment and subsequent assessment. Even in this update, a few new Perl and Python scripts were included in the pipeline for the preparation of files and removal of outliers. However, there were a few steps where manual intervention was still required for producing better alignment. The superfamily member(s), which showed >5.5 Å RMSD with other members, or the ‘structural outliers’, have been listed in a text file named ‘struc_outliers’ under each superfamily (Supplementary Figure 2). The RMSD values are provided in the database for the members considered for alignment for all MMS.

**Figure 3:**
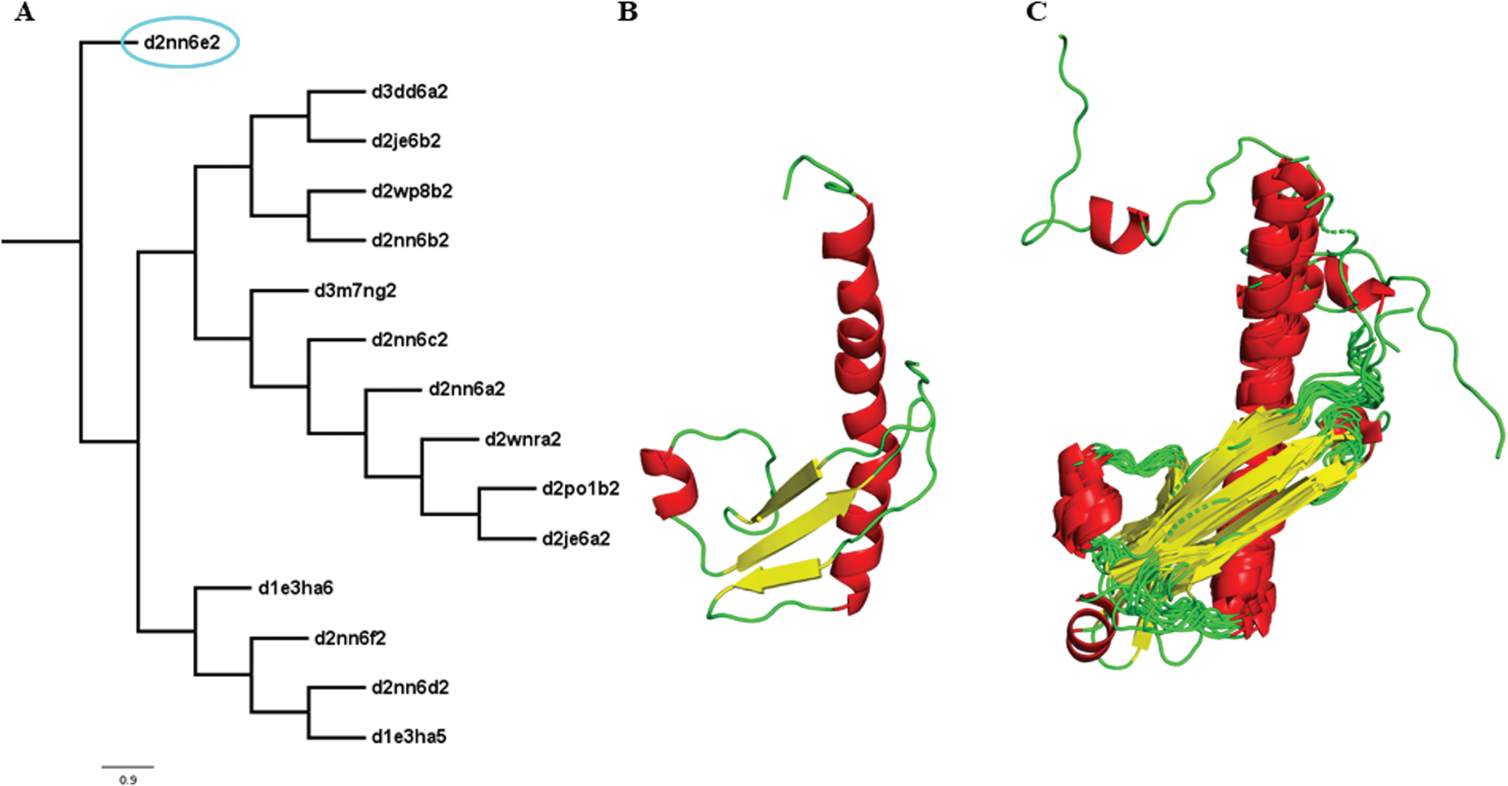
Illustration of a PASS2 superfamily with an extreme structural outlier domain. (**A**) Structural phylogeny of the members of the Ribonuclease PH domain 2-like superfamily (SCOPe superfamily ID: 55666) showing extreme structural outlier (d2nn6e2, circled in cyan). (**B**) Structure of the extreme structural outlier, d2nn6e2, and (**C**) the final superposed structure of remaining members after removal of extreme structural outlier.

Two new concepts have been introduced in this pipeline and version: extreme structural outliers and split superfamilies. ‘Extreme structural outliers’ (Figure 3) are domains that fail to align with any other member of a particular superfamily, and were identified for each superfamily by examining the intermediate files generated by COMPARER as a result of superposition, and checking for insufficient number (less than 3) of equivalences. Such extreme structural outliers, if any, were removed and have been provided in the ‘outlier’ text file under each superfamily. The remaining members of the superfamily were realigned using the protocol detailed in Figure 2. For a few superfamilies, the superfamily definition was redefined to split superfamily, as equivalences could not be extracted. Such splitting of superfamily was guided by manual inspection of the COMPARER alignment and the structural dissimilarity-based phylogeny (Supplementary Figure 3). Similarly, when dealing with large superfamilies, extraction of equivalences proved difficult for few of them and the superfamilies were split in the same way to provide a better alignment. In few cases, where COMPARER-based alignment was rendered difficult due to multiple reasons, a preliminary structure-based alignment (using Matt algorithm) has been provided for download, along with corresponding structural annotations.

### Mapping functionally important residues and HCR

Protein domains belonging to the same superfamily are usually sequentially divergent, but they share common structural and functional features, by the virtue of having a common ancestor (19). It is often observed that families belonging to the same superfamily often have additional motif(s) and/or distinct residue patterns within motifs that are involved in substrate specificity or family-specific biological functions. Such family-specific functionally important residues (FIRs) can be identified using sequence alignment of the superfamily members. In the superfamily CoA-dependent acyltransferases (CAT) (SCOPe superfamily ID: 52777), FIRs His, Asp and Gly form the catalytic motifs (HXXXD and HHXXXDG), which correspond to two SCOPe families—CAT-like family (SCOPe family 52778) and Non Ribosomal Peptide Synthetases (NRPS) condensation domain (amide synthase) (SCOPe family 75229), respectively (Supplementary Figure 4) (20).

The Interleukin 8-like chemokine superfamily (SCOPe superfamily ID: 54117) belongs to the α + β class and contains two families in SCOPe. The homologues of Interleukin-8 include small cytokines or signalling proteins secreted by the cell during inflammation and are together referred as chemokines. The structure-based sequence alignment of the members of this superfamily revealed four conserved Cys residues, which are known to form two disulphide bonds. Members of Interleukin 8-like chemokine superfamily are further classified into two subfamilies, CXC and CC on the basis of the motif patterns involving two N-terminal Cys residues. There are, however, two additional branches of subfamilies CX3C and C-type, which have domains with two Cys separated by three X or non-conserved amino acid positions and only a single residue of Cys, respectively (Supplementary Figure 5) (21, 22).

### Features of the alignments available for download

Structure-based sequence alignments can be used as reliable evolutionary models for large-scale sequence searches and prior knowledge of motifs, which are signatures of the superfamily may further aid the search. HMMs of alignments of superfamily members and conserved secondary structural motifs (in PROSITE format) have been provided in the database. HCRs have been mapped onto the superfamily alignments and can be used for rational design of structure–function test experiments, such as mutational analyses. The alignments were annotated to describe solvent accessibility, hydrogen bonding patterns and secondary structural features. PCA was performed for superfamilies, based on sequence similarity distribution of members of a superfamily, and the PCA plots are available for download in both PDB and PDF formats. Other than these, alignment statistics (Alistat) and insertion–deletion and length variation information (CUSP) have been provided for each superfamily. A structural phylogenetic tree of the protein structural domains (based on C^α^ RMSD) is available for download for each superfamily. The percent sequence identity and the C^α^ RMSD-based distance matrices are present in downloadable format. The mean C^α^ RMSD values of the domains within each superfamily have also been provided as ‘MeanRMS’ files for download. Each superfamily member has been mapped to GO term(s) to indicate the most probable functions associated with the protein chains and/or domains in a superfamily.

## Conclusion

Homologous proteins are related by sequence, structure and function. However, divergent evolution leads to diminishing sequence identities among homologues, with retention of structural topology and function. Since structure is better conserved in evolution than sequence, structure-based sequence alignments have been commonly used as the gold standard for sequence alignment evaluation. Availability of reliable sequence alignments for distantly related proteins within a superfamily are essential for homology searches, building three-dimensional structural models of proteins and understanding structure–function relationships. PASS2 database provides such structure-guided sequence alignments for superfamilies listed in SCOP (2–4, 23–25). The first update of PASS2 in 2002 was in correspondence with SCOP 1.53. The number of superfamilies and domains considered for structure-based sequence alignment in PASS2 have also increased consistently, with deposition of newer structures in PDB and classification in SCOP (2). PASS2 has been used for large-scale sequence searches, structure-based phylogenetic analyses of protein domain superfamilies, analysis of structure-based sequence alignment methods and many others (23, 26, 27).

The current update of PASS2, PASS2.6, provides structure-based sequence alignment of members of superfamilies (with no more than 40% sequence identity within themselves) and is in accordance with the latest release of SCOPe, i.e. SCOPe 2.06. Apart from alignments, multiple features including HMMs, structural motifs, GO annotations and HCRs have been provided, which can aid further studies on the protein domain superfamilies such as genome-wide sequence searches and design of mutational analysis-based experiments. In this update, we have taken into account extreme structural outliers and split superfamilies, where required. In cases where COMPARER based alignment was rendered difficult, initial Matt alignment has been provided along with accessory files for the superfamily. PASS2.6 is available at http://caps.ncbs.res.in/pass2/.

## Supplementary Material

Supplementary_Figures_revised_final_baz028.doc

## Abbreviations

ACR, absolutely conserved residue
FIR, functionally important residue
GO, Gene ontology
HCR, highly conserved residue
Matt, Multiple Alignment with Translations and Twists
MMS, multi-member superfamily
PASS2, Protein Alignments organised as Structural Superfamilies
PDB, Protein Data Bank
RMSD, Root Mean Square Deviation
SCOP, Structural Classification of Proteins
SMS, single-member superfamily
PCA, Principal Component Analysis.

## Declarations

All authors have gone through the manuscript and contents of this article have not been published elsewhere.

## Notes

**Database URL**: caps.ncbs.res.in/pass2
