# Supplementary material for: PASS2 version 6: a database of structure-based sequence alignments of protein domain superfamilies in accordance with SCOPe
Source: Database (Oxford). 2019 Mar 1;2019:baz028. doi: 10.1093/database/baz028 (PMC6395796; doi:10.1093/database/baz028)
Supplement: Supplementary_Figures_revised_final_baz028.doc [file supplementary_figures_revised_final_baz028.doc]

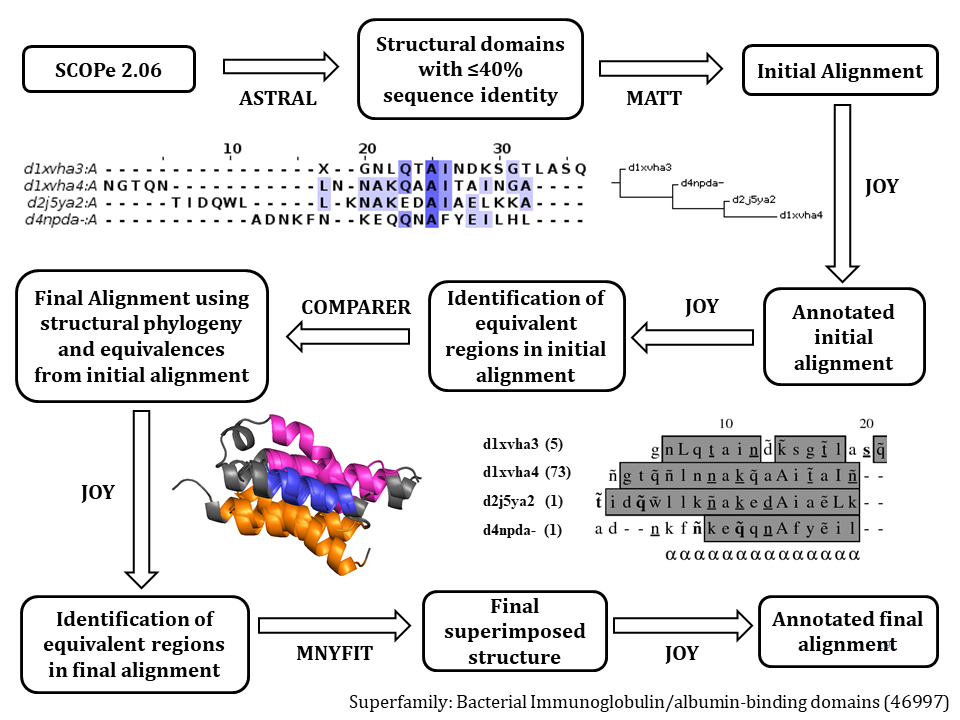


Supplementary Figure 1: Detailed workflow of PASS2.6, showing initial alignment of members of the Bacterial Immunoglobulin/albumin-binding domain superfamily (SCOPe superfamily ID: 46997). Initial Matt derived alignment and RMSD-based structural phylogenetic tree are shown. Three equivalent regions or equivalences, shown on the superposed structure with colours orange, blue and magenta were used to obtain the final COMPARER alignment which has been annotated using JOY.


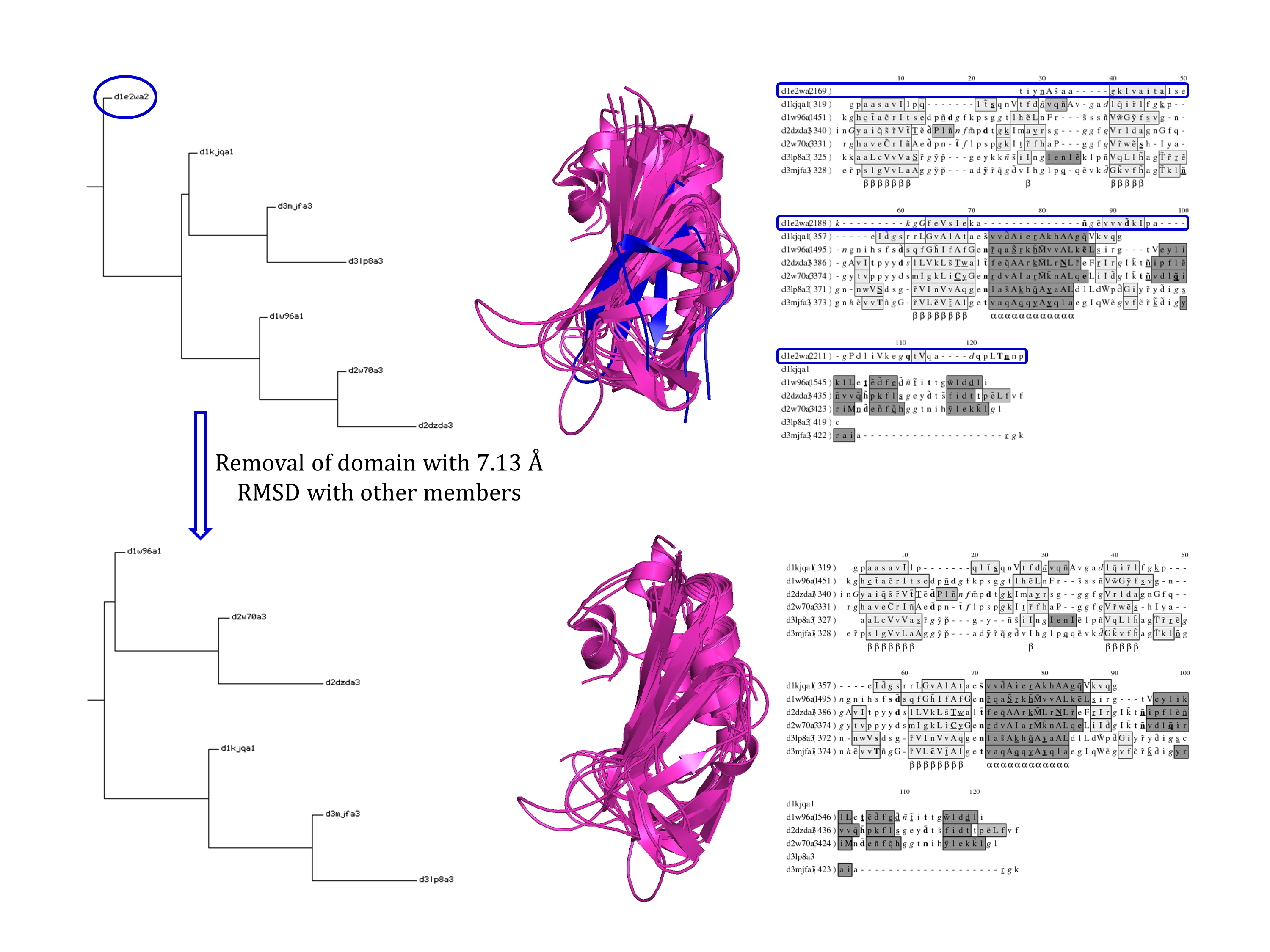


Supplementary Figure 2: For the superfamily Rudiment single hybrid motif (51246), the domain d1e2wa2 (coloured in blue, corresponds to N168F mutant of Cytochrome f) showed 7.13 Å RMSD with other members and was removed as structural outlier. It also appears deviant in the structure-based sequence alignment, with an insertion (marked in blue box).


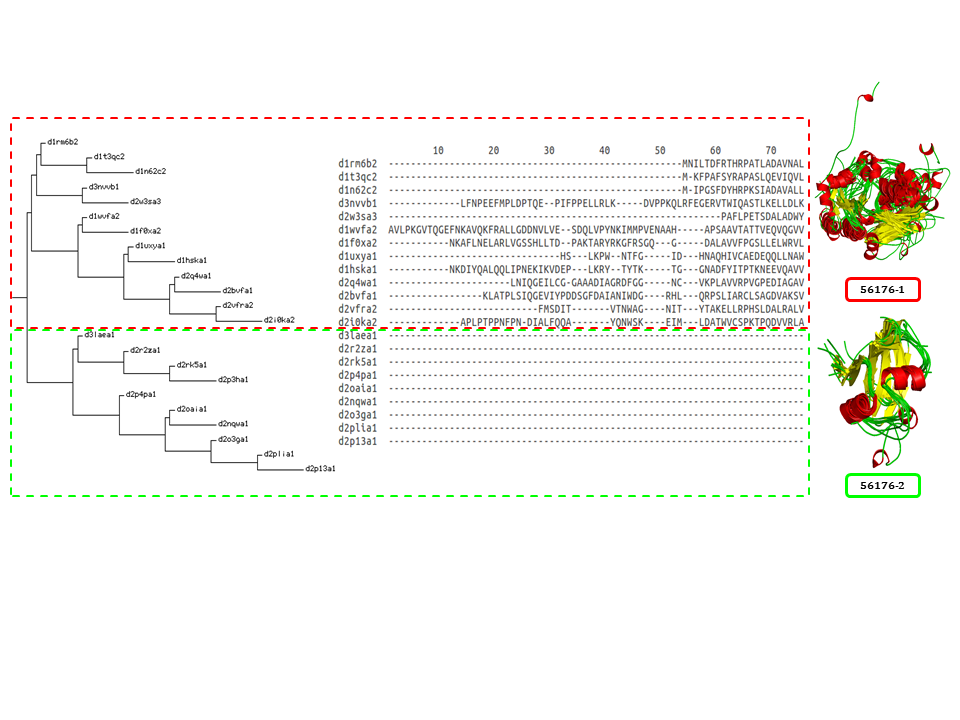


Supplementary Figure 3: The members of the FAD-binding/transporter-associated domain-like superfamily (56176) form two clusters in the RMSD-based structural phylogeny (left). This is also seen in the COMPARER-derived alignment (middle). The superfamily members were split accordingly and aligned separately. The final superposed structures of each the split superfamilies is given in the right, coloured according to secondary structure.


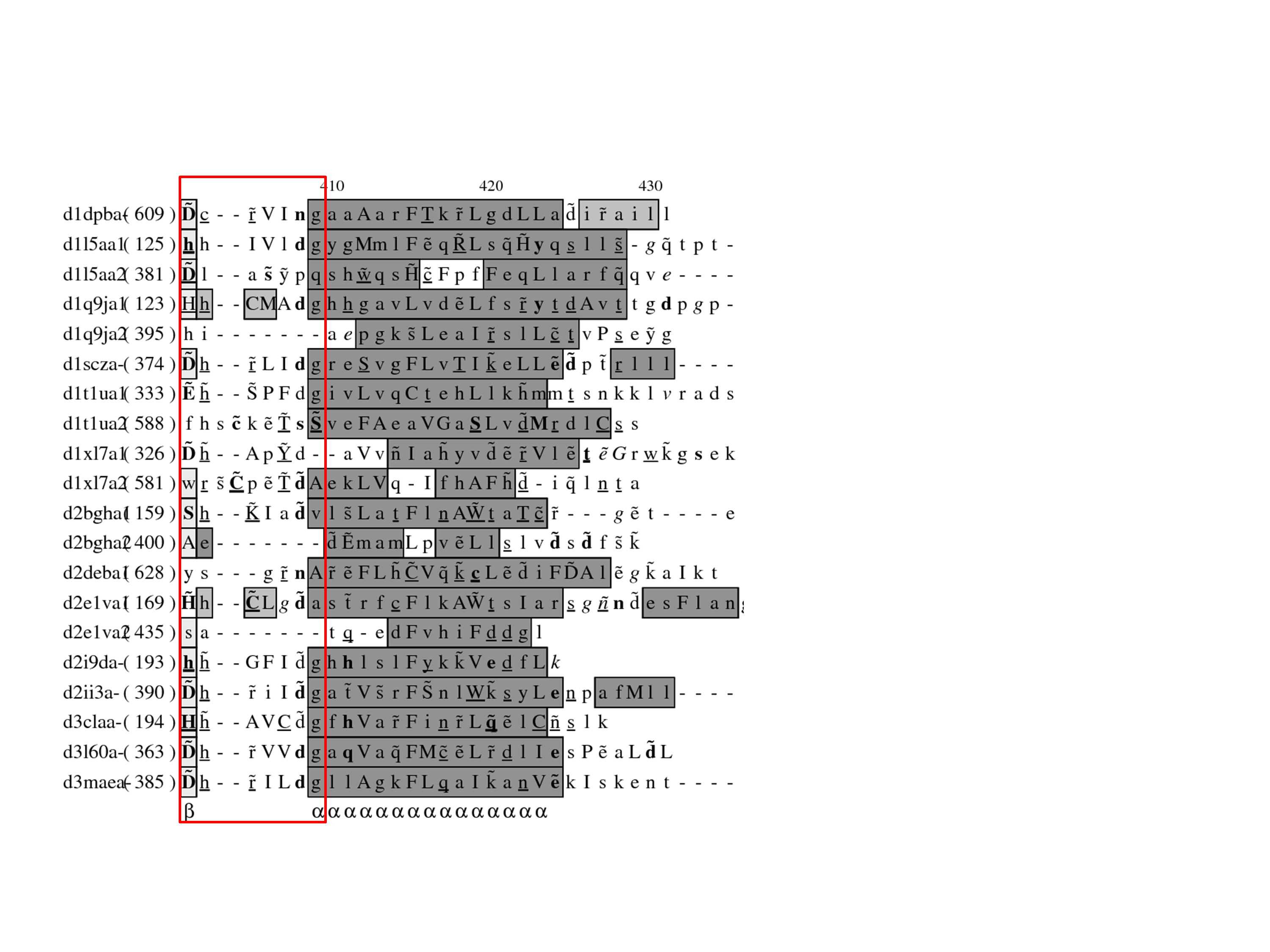


Supplementary Figure 4: JOY annotated structure-based sequence alignment of the members of the superfamily- CoA-dependent acyltransferases (52777) showing functionally important residues His, Asp and Gly forming the catalytic motif (red box) HXXXD and HHXXXDG in the families CAT-like and NRPS condensation domain (amide synthase).


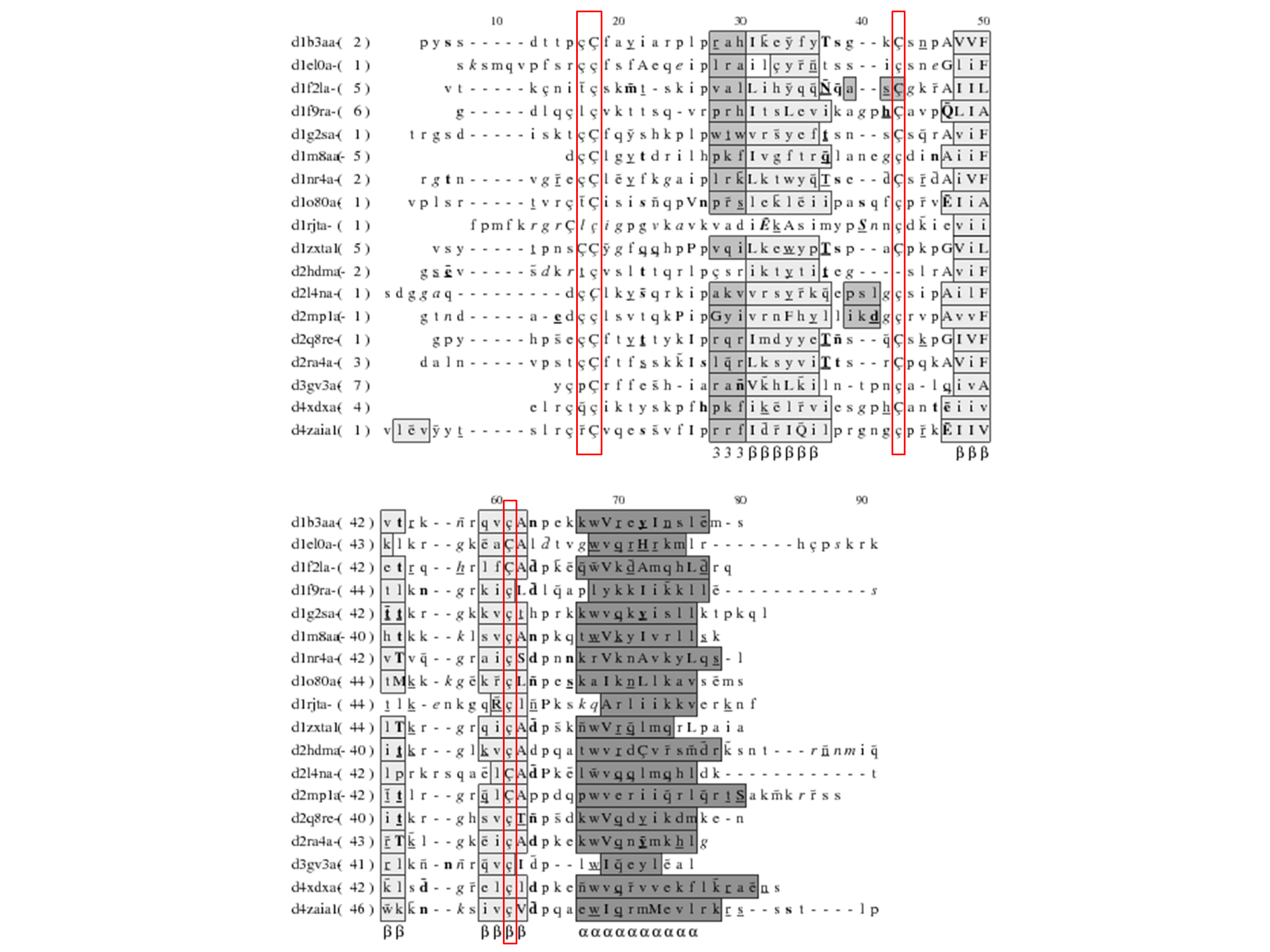


Supplementary Figure 5: JOY annotated structure-based sequence alignment of members of the Interleukin 8-like chemokine superfamily (54117) showing four Cys residues (red boxes) which form two disulphide bonds and are conserved throughout the superfamily as seen in the alignment.
